# Comparison of inhaled versus intravenous anesthesia for laryngoscopy and laryngeal electromyography in a rat model

**DOI:** 10.1186/s40463-018-0312-9

**Published:** 2018-10-20

**Authors:** M. Gazzaz, J. Saini, S. Pagliardini, B. Tsui, C. Jeffery, H. El-Hakim

**Affiliations:** 1 Division of Otolaryngology-Head and Neck Surgery, Department of Surgery, University of Alberta, 2C3.57 Walter MacKenzie Centre, Edmonton, AB T6G 2R7 Canada; 2 Neuroscience and Mental Health Institute, University of Alberta, Edmonton, AB Canada; 3 Women and Children Research Institute, University of Alberta, Edmonton, AB Canada; 4 Department of Physiology, University of Alberta, Edmonton, AB Canada; 5 Stanford University Pediatric Regional Anesthesia, Stanford University, Stanford, California USA

**Keywords:** Laryngeal mobility disorders, Laryngeal electromyography, Inhalational anesthesia, Total intravenous anesthesia

## Abstract

**Background:**

Propofol and remifentanil intravenous combination is one popular form of total intravenous anesthesia (TIVA) in mainstream clinical practice, but it has rarely been applied to a rat model for laryngoscopy and laryngeal electromyography (LEMG). Our objective was to establish a safe and reproducible general anesthetic protocol for laryngoscopy and endoscopic LEMG in a rat model. Our hypothesis is that TIVA allows a minimally morbid, and feasible laryngoscopy and LEMG.

**Methods:**

Sprague Dawley rats were subjected to either inhalational anesthesia (IA) (isoflurane) or TIVA (propofol and remifentanil) and underwent laryngoscopy and LEMG. The primary outcome was a complete minimally interrupted rigid laryngoscopy and obtaining reproducible motor unit potentials from the posterior cricoarytenoid muscles. The secondary outcome was morbidity and mortality.

**Results:**

Seventeen out of twenty-two rats underwent both TIVA and IA. Only two underwent IA only. All nineteen rats that underwent IA had a successful experiment. Seventeen rats underwent TIVA, however, only nine completed a successful experiment due to difficulty achieving a surgical plane, and respiratory events. Upon comparing the success of the two anaesthetic regimens, IA was superior to TIVA (*P* = 0.0008). There was no statistical difference between the amplitudes (*p* = 0.1985) or motor units burst duration (*p* = 0.82605) of both methods. Three mortalities were encountered, one of which was due to lidocaine toxicity and two were during anesthetic induction. Respiratory related morbidity was encountered in two rats, all seen with TIVA.

**Conclusions:**

TIVA is not an ideal anesthetic regimen for laryngeal endoscopy and LEMG in rat models. Contrary to our hypothesis, IA did not affect the quality of the LEMG and allowed a seamless rigid endoscopy.

**Electronic supplementary material:**

The online version of this article (10.1186/s40463-018-0312-9) contains supplementary material, which is available to authorized users.

## Background

The standards of general anesthesia for airway endoscopy in humans have evolved due to developing technological and pharmacological innovations. One of the most challenging diagnoses to establish in laryngology is mobility disorders, particularly in children where endoscopy under general anesthesia, supplemented accordingly by laryngeal electromyography (LEMG) is the reference standard.

Traditionally, inhalational anesthesia (IA) was routinely employed for airway endoscopy in clinical practice. In the late 1990’s, total intravenous anesthesia (TIVA) technique was introduced and gained popularity [1]. But to this day, proponents of both options argue their cases strongly. The claimed advantages of IA include speed, ease and comfortable induction using a mask in the absence of intravenous (IV) access, in addition to simple non-invasive evaluation of the blood tension of the inhaled agent [2]. On the other hand, TIVA is professed to lessen postoperative nausea and vomiting, act rapidly and independently from the alveolar ventilation, and is administrable using peripheral locations away from airway instrumentation. It is also a non-pollutant for the operative room environment [2–4].

Some experts in airway endoscopy and electromyography studies support the notion that anesthetic agents may modify the findings. It is proposed that different concentration and duration of IA may modify LEMG findings and produce spurious abnormalities. Some rest this notion on evidence from literature pertaining to spine surgery [5–8]. However, many centers perform LEMG in humans under IA [9–12], especially that there are no head to head studies comparing the two techniques. In rats, LEMG has also been performed under IA [13], yet in most cases it has been used as an induction agent for sedation [14–17].

Propofol and remifentanil IV combination is one popular form of TIVA in mainstream clinical practice, but it has rarely been applied to a rat model for laryngoscopy and LEMG. We therefore set out to evaluate whether a TIVA protocol is applicable to the rat model for reproducible assessment of laryngeal function, with minimal morbidity. We specifically aimed to compare the mortality, morbidity, and reproducibility of two general anesthetic protocols for laryngoscopy and endoscopic LEMG in a rat model. Our hypothesis was that TIVA allows a minimally morbid, and feasible laryngoscopy and LEMG.

## Methods

### Study design

The experiment was conducted in accordance with the Canadian Council of Animal Care guidelines and policies, following approval from the University of Alberta Health Research Ethics Board (AUP00001311) and the Animal Care and Use Committee for health sciences at the University of Alberta.

This prospective comparative non-randomized, cross over experimental animal study was conducted at the Surgical Medical Research Institute and Katz Group - Rexall Centre for research at the University of Alberta, between April 2016 and February 2017.

After induction of general inhalation anesthesia using isofluorane (2% in air; IA) to set up vein cannulation, anesthesia was maintained under one of the two anesthetic options followed by the second one (i.e TIVA followed by IA or vice versa) allowing a washout period to occur in between, during which the animal shows a positive toe pinch reflex.

### Study subjects

A total of 30 Sprague-Dawley rats were approved for this study. All rats were housed in pairs within the housing facility of Health Sciences Laboratory Animal Services at the University of Alberta. Eight rats were used initially as a pilot study.

### Experimental procedure

#### Preparation and anesthesia

Pre-operatively, age, sex, and weight of the rats were documented and a unique identifying number was given. Rats were then placed in an induction chamber saturated with 2% isoflurane. Anesthesia was maintained using either inhaled isoflurane 1.5–5% or a combination of propofol (10 mg/ml and 40–50 mg/kg/h IV infusion) [18] and remifentanyl (5 mcg/ml and 0.4 mcg/kg/min IV infusion) [19] after establishing IV access (either via tail or femoral vein). Ampicillin (50 mg/kg SC), meloxicam (1-2 mg/kg SC) and ringer’s lactate (1 ml/kg/hr. intraperitoneal) were administered preoperatively. The rat was then transferred to the surgical table and placed on a restraining board with an integrated circulating fluid heating pad with temperature set at 37 °C. A respiratory belt (Kent Scientific Co., USA), a rectal thermometer probe and a vital signs monitoring foot sensor (STARR Life Sciences® Mouse Ox® Plus) were attached. The depth of anesthesia was determined by eliciting a toe pinch reflex, observing the respiratory rate and pattern of breathing, and finally the tolerance and response to airway stimulation to endoscope insertion. If TIVA was used for maintenance, isoflurane concentration was reduced to 0.5%, and then turned off after a period of five minutes. The depth of the anesthesia was then assessed again and the rate of infusion was adjusted accordingly. Baseline and periodic readings of heart and respiratory rate, peripheral capillary oxygen saturation (SpO2), temperature and mucous membrane color were all recorded every five minutes.

#### Laryngoscopy and laryngeal EMG

Once the rat was adequately anesthetized under either IA or TIVA, room air (21% O2) was delivered through the nasal mask for 1–2 min to maintain SpO_2_ above 90%. The rat was then positioned supine on the experimental workstation inside a Faraday cage. By retracting the tongue, the larynx was visualized and lidocaine 1% (1.67 mg/kg) was applied topically under telescopic guidance. A nebulizer was also connected to the nasal cone and lidocaine 1% was delivered for ~ 1 min. This step was aborted in future experiments after presumed lidocaine toxicity mortality was encountered.

#### Laryngoscopy

While the animal was spontaneously breathing, a zero degree 2.7 mm rod lens telescope (KARL-STORZ®, Germany) connected to an image capture unit, was used to visualize vocal cords’ movements.

#### Laryngeal electromyography

Once the larynx was exposed, LEMG recordings from the posterior cricoarytenoid (PCA) muscle were obtained by inserting a monopolar needle electrode (29GA, 37 mm) (Rochester Electro-Medical, USA) transorally under direct rigid endoscopic visualization with each anesthetic regimen. Since the PCA muscle is responsible for vocal cord abduction during inspiration, in addition to the ease of electrode insertion in comparison to other adductor intrinsic laryngeal muscles in the tenuous rat airway, we elected to choose it as our muscle of choice to obtain LEMG recordings. A ground electrode (27G, 12 mm) (Ambu® Neuroline Subdermal, Malaysia) was secured into the chest. Electrodes were connected to amplifiers (AM Systems, Carlsborg, WA) and activity was filtered between 300 Hz and 1 kHz, amplified at x10k and sampled at 1 kHz (Powerlab 16/30; AD Instruments, Colorado Springs, CO). A piezoelectric chest belt was connected to the recording system in order to detect chest wall movements and correlate between LEMG signal and the respiratory cycle. A minimum of ten respiratory cycles was digitally recorded from the muscle for off-line analysis.

#### Recovery, postoperative care and euthanasia

Upon the conclusion of the experiment the rat was transferred to a new cage to allow recovery from anesthesia. Each rat was housed individually for 2 h post-operatively to be monitored and assessed clinically every fifteen minutes. This included activity, response to external stimuli, appearance and feeding. The animal was then euthanized by decapitation under isoflurane anesthesia.

### Outcome measures

#### Primary outcomes: Proportion of complete rigid laryngoscopy and LEMG

A successful experiment was defined as completion of both laryngeal endoscopy and the ability to obtain a reliable LEMG recording. A complete laryngoscopy was defined as a minimally interrupted, well-tolerated rigid endoscopy of the respiratory action of the larynx while the subject goes through ten cycles of spontaneous breathing. For LEMG, ten consecutive respiratory related bursts of activity were required and the mean amplitudes and burst durations of the LEMG signal were analyzed and calculated using Lab ChartPro8.

Criteria used to abort the experiment included: signs of hemodynamic instability encountered during intraoperative monitoring (persistent maximal scores of respiratory distress, i.e. apnea/hypopnea, or sustained heart rate deviations) or a ninety minutes maximum duration as a cut-off point to achieve the appropriate depth of anesthesia.

#### Secondary outcomes: Mortality and morbidity

Mortalities encountered were documented. Morbidities experienced were defined as respiratory events during the procedure, which included laryngeal spasm, apnea and hypopnea requiring interruption of the procedure.

### Statistical analysis

Demographics were summarized as means, standard deviation (SD), minimal and maximal values. Student t-test was used for comparing means, and 95% confidence intervals were provided. Fisher’s exact test and chi square were used to compare proportions of mortality and morbidity between anesthetic regimens [20].

Based on a previous study from our laboratory using only propofol as TIVA [18], a 70% respiratory morbidity rate was demonstrated. A decision was made that a reduction to 15% would be statistically and clinically significant. Based on 16.67% mortality rate and accepting a *p* value of 0.05 and a power of 80%, the sample size would be ten per group. Allowing for unforeseen morbidity, five rats were added to each group for a total of 30 rats.

As part of the LEMG waveform evaluation, we included motor unit potential assessment in the form of amplitude and burst duration. The amplitude was calculated from the height, the burst duration from the period and 60 divided by the period to obtain the respiratory rate using peak analysis in Lab ChartPro8.

## Results

A total of 30 rats were used. The mean age was 7.56 ± 5.79 months (3–18). Thirteen were males and seventeen were females. The mean weight was 509.02 ± 258.24 g (245–1200). The basic clinical parameters for the two groups are described in Table 1. All 8 rats from the pilot group underwent TIVA and two of them underwent a successful experiment.

**Table 1:** Parameters of the rats

Parameter	TIVA (*n* = 17)	IA (*n* = 19)
Age (months) Mean ± SD (range)	7.18 ± 6.1 (3–18)	6.74 ± 5.9 (3–18)
Males	9	9
Females	8	10
Weight (gm) Mean ± SD (range)	514.88 ± 219.28 (245–980)	488 ± 217.95 (245–980)

**Fig. 1:**
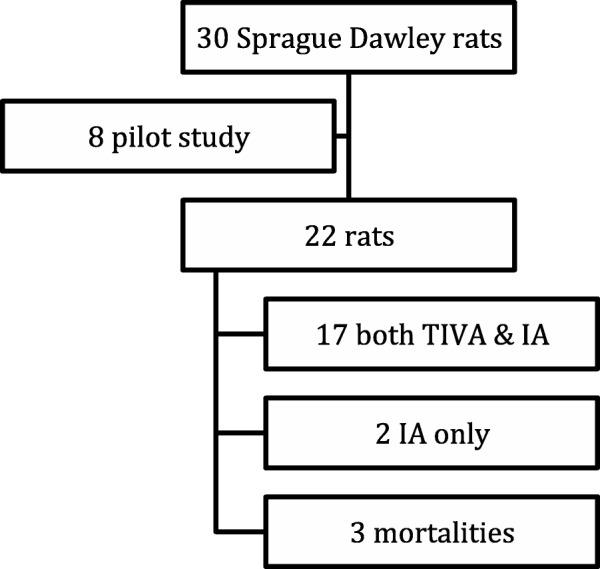
General scheme of the study

Seventeen (77.3%) rats underwent both TIVA and IA, two (9.1%) underwent IA only. Three (13.6%) mortalities were encountered in total. Two of them occurred during anesthetic inhalational induction and one mortality was likely due to lidocaine toxicity while on IA. Eight rats (47.06%) were maintained with TIVA first followed by IA and twelve (52.94%) were maintained with IA first followed by TIVA. See Fig. 1.

**Fig. 2:**
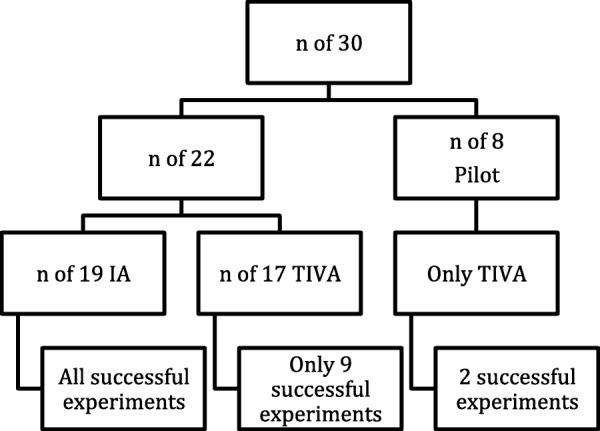
Successful experiments under TIVA and IA

All nineteen rats that underwent IA had a successful experiment requiring a maximum period of 15 min, i.e. tolerated endoscopy without major respiratory events and completed a reproducible LEMG. Out of the 17 rats that underwent TIVA, nine of them (52.94%) completed a successful experiment requiring a duration between 45 and 90 min. See Fig. 2. The eight unsuccessful experiments (47.06%) were mainly due to inability to achieve an appropriate anesthetic plane. Seven of these rats continued to be responsive and intolerant of endoscopy despite escalation of the TIVA dosage to as high as 3.5 times the weight and boluses, whereas one animal developed bradycardia down to 70 beats per minute and the SpO_2_ dropped to 60%, and the procedure was aborted to ensure safety. In the nine successful experiments under TIVA, no mortalities were encountered. However, two rats (11.76%) developed apneic events for seconds during the procedure and recovered spontaneously.

While the rats were maintained on IA, no morbidities were encountered. Only one (5.26%) mortality took place (presumably due to lidocaine toxicity). Comparing both anesthetic regimens, no statistical significance was evident for morbidity (*p* = 0.096) or mortality (*p* = 0.679), however, IA proved to be superior to TIVA in performing successful experiments (*p* = 0.0008).

With regards to LEMG variables, it was noted that the mean amplitude of LEMG in TIVA is 66.9% that of IA. However, there was no significant difference between mean amplitudes − 1.79 ± 9.88 mV (95% CI -1.79-2.2, *p* = 0.1985) or mean burst duration 0.27 ± 0.75 s (95% CI -0.23-0.76, *p* = 0.82605). See in the Additional file 1: Table S1 for details.

**Fig. 3:**
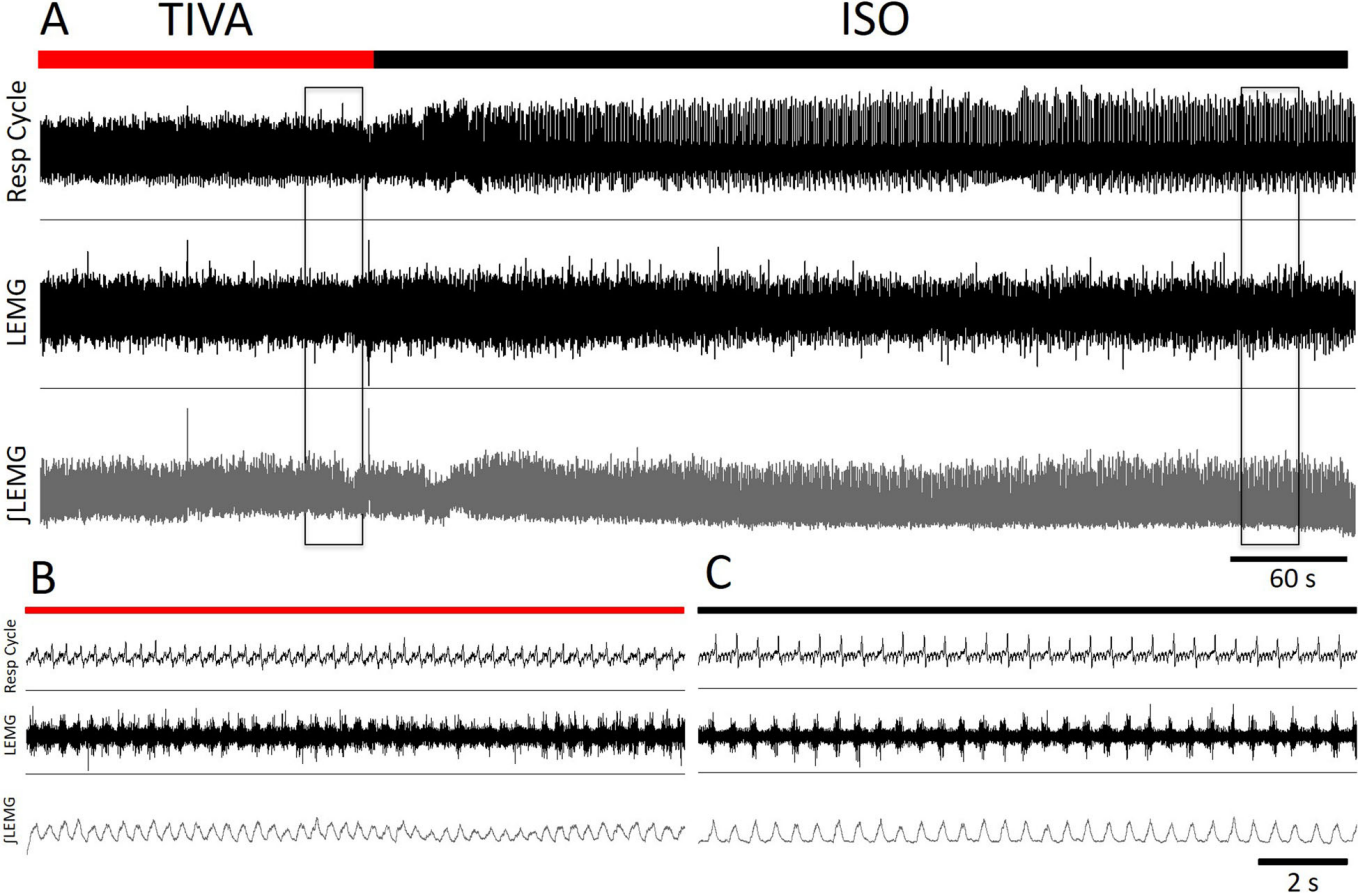
Respiration and LEMG recordings. Respiration and LEMG recording from PCA muscles under TIVA (Red) and isoflurane IA (Black). **a** PCA LEMG recording from a rat initially under TIVA anesthesia and transitioning to IA. Recordings include chest belt measurement to determine respiratory activity (top), the raw LEMG measurement from the PCA muscles (middle) and the integrated LEMG signal (bottom). **b** A 30 s inset of the recording under TIVA. **c** A 30 s inset of the recording under isoflurane

Two rats had the electrode maintained in the same position without manipulations while the anesthetic regimens were switched. No statistical difference in mean amplitude or mean burst duration was evident individually (*p* > 0.05) despite the amplitude being lower on TIVA. The PCA contraction displays a pre-inspiratory pattern of activity in both IA and TIVA consistently. See Fig. 3.

Of note, the time required to perform a full experiment using IA was 10–15 min compared to 45–90 min when using TIVA.

When comparing clinical parameters during the recovery period, no morbidities or mortalities occurred during recovery.

## Discussion

This study compared the use of TIVA and IA during laryngoscopy and LEMG recording in rat models. Our endpoints were the ability to perform a complete endoscopy and neurophysiological recordings with the least morbidity and mortality. Our results show that TIVA is unlikely to be the anesthetic of choice for endoscopy and LEMG recordings in a rat model.

Balancing adequate depth of anesthesia and stability of spontaneous respiration during pediatric endoscopic surgery may be difficult to maintain using TIVA [21, 22]. Additionally, drug dosing appears more demanding with TIVA and higher infusion rates are sometimes necessary to provide the desired plasma concentrations [2]. Malik and Sen [23] reported 5.3% respiratory related morbidity manifested as brief episodes of desaturation due to malposition of the airway and laryngospasm with intermittent TIVA for pediatric endoscopic procedures. On the other hand, evidence suggests that TIVA reduces airway reactivity; decreases bronchospasm and laryngospasm in children [4, 24].

In the current study, the morbidity rate was reduced from 70% based on previous experiments performed by the senior author HE [18] to 11.7% which is considered clinically significant in the current experiment. This may be due to the effect of adding remifentanyl.

Several studies have compared different types of anesthetics regimes in pediatric otolaryngology surgeries. A direct comparison between TIVA (propofol plus remifentanil) and volatile anesthetics indicated that TIVA is superior for induction, maintenance and recovery from anesthesia in children undergoing flexible fiberoptic bronchoscopy, adenoidectomy and/or tonsillectomy [25, 26].

Evidence in pediatric IA suggests that it may cause apnea following induction, especially if great concentrations were delivered [2]. This may explain the unexpected mortalities encountered during induction under IA in our current study.

Several anesthetics in experimental animal models have been used to perform safe, interpretable and reliable endoscopy and/or LEMG, but direct comparisons and proof of reproducibility are limited [13–17, 27–32]. These included intraabdominal barbitone sodium [27], mixture of intramuscular ketamine hydrochloride and xylazine hydrochloride [14, 15, 30] intramuscular ketamine only [28], combination of inhaled isoflurane and intraperitoneal ketamine and xylazine [16, 17, 29, 31], intraperitoneal/intravenous pentobarbital sodium [32], and isoflurane only [13]. With the exception of ketamine and isoflurane, none of these drugs are used in clinical practice. Interestingly, only scant reports on mortality and morbidity have been previously described given the fragility of the animal and its delicate airway, with prior experience indicating up to 20% mortality [13, 18, 33, 34].

Our results indicate that LEMG can be reliably performed under IA in a rat model. Despite previous studies in human spinal surgery [5–8] and animal studies [35] suggesting that the duration and concentration of IA affect evoked electromyogram parameters, specifically amplitude and latency, our results showed no difference in spontaneous LEMG variables between anesthetics. Several LEMG clinical studies [36–39] have used TIVA instead of IA when studying laryngeal disorders. The exact reasons are unclear, perhaps due to the increased use of TIVA among pediatric anesthetists or the potential effect of IA on LEMG.

One important observation in our study is the reduction in the duration of a complete experiment using IA (10–15 min) compared to TIVA (45–90 min). The advantages of using IA compared to TIVA in terms of reduction in induction time, maintenance of stable breathing, lack of laryngeal bronchospasm and emergence from anesthesia has been also reported previously in pediatric studies [21, 40]. In our study, the longer duration required while on TIVA was mainly due to inability to achieve an appropriate anesthetic plane to perform the experiment. This may well be due to IV agents displaying excessive inter-individual variability to TIVA maintenance that cannot be easily estimated [2] or perhaps associated with the large body weight of some rats used for this study and the accumulation of fat mass that may alter the dose necessary to establish the optimal surgical plane.

Tsai and colleagues [41] compared the recovery from laryngoscopy procedures under propofol TIVA and conventional isoflurane in a canine model. The TIVA group was significantly better than the isoflurane group in terms of smoothness of recovery from surgery, defined as absence of struggling, vocalization, or excitement and requiring little or no physical restraint to prevent self-injury. However, the isoflurane group recovered faster from anesthetic. No significant difference was observed between the two groups in terms of adverse effects, which was comparable to our findings.

LEMG is considered a valuable clinical and research tool to assess different pathologies in laryngeal motor function. Its’ use has been described in the literature for humans and in experimental models as an outcome measure following a specific laryngeal intervention. As part of the reporting practice, the type of anesthetic used throughout the procedure should be documented [42]. Proponents of LEMG argue its prognostic and diagnostic values that may guide treatment decisions in patients with vocal fold mobility disorders [37, 43].

Multiple intrinsic laryngeal muscles were described in the literature as a point for recording LEMG activity in both animals and humans. This includes thyroarytenoid [9–12, 15–18, 33, 36–39, 44–49], cricothyroid [44, 46, 48, 50], PCA [11–13, 15, 16, 18, 27, 32, 33, 37, 38, 44, 45, 49, 51, 52] and lateral cricoarytenoid [15] either individually or in combination. The authors have selected the PCA muscle, as it’s the single laryngeal muscle responsible for vocal fold abduction. PCA contraction has been pre-inspiratory on a consistent basis noted by the piezoelectric chest belt in our study.

One of the study limitations is the wide range of weight (245 g–980 g) and the large weight potentially contributing in the rat’s morbidity and/or mortality while under general anesthesia. Despite the known typical weight of laboratory rats ranging between 300 g and 500 g, mortalities encountered were amongst smaller animals weighing equal to or less than 400 g and morbidities were seen within the mean of weight.

We acknowledge that the monopolar needle electrode was inserted in different locations within the PCA muscle with each anesthetic regimen, which may have affected the LEMG recordings. We are aware that recordings depend on the size of muscle fibers, the proximity of the electrode to large muscles, and the depth of electrode insertion. However, maintaining the electrode in the same position is practically difficult to achieve. A proper washout period between the two anesthetic methods cannot be feasible. Still, we were able to keep the electrode in the same location without manipulation in 2 rats and found no statistical difference in mean amplitude or burst duration.

An additional limitation is the fact that the rats were not blindly randomized for the anesthetic regimens, nor were the results concealed due to the nature of the experiment. However, we used a cross over trial instead. In such case, randomization or which anesthetic was started first does not matter, as the rat will undergo both anesthetic regimens regardless. Also, TIVA and IA are known to have rapid onset/offset action, the effect of anesthetic is reversible, the period of administration is short; the condition is relatively stable as the rats were completely healthy, and the carry-over is not an issue [53]. This permitted convenience and efficiency of the project [54]. This design also allowed amplification of the sample size used.

In the future, we aim to replicate the same experiment in pediatric patients and compare anesthetic regimens and their effect on laryngoscopy and LEMG, as IA is a useful translational model for laryngoscopy and LEMG experiments.

## Conclusion

Contrary to our hypothesis, IA did not affect the quality of LEMG and allowed a seamless rigid endoscopy in rat models superior to TIVA. It proved to be quick, easy and safe to administer. We conclude that this is a reliable translational model for laryngoscopy and LEMG experiments.

## Additional file

Additional file 1: **Table S1.** LEMG parameters. (DOCX 61 kb)

## Abbreviations

IA: Inhalational anesthesia
IV: Intravenous
kg/min: Kilogram/minute
L/min: Liters/minute
LEMG: Laryngeal electromyography
mcg/kg/min: Microgram/kilogram/minute
mcg/ml: Microgram/milliliter
mg/kg: Milligram/kilogram
mg/ml: Milligram/milliliter
min: Minutes
ml: Milliliter
ml/kg/hr: Milliliter/kilogram/hour
mm: Millimeter
O_2_: Oxygen
PCA: Posterior cricoarytenoid
SC: Subcutaneous
SD: Standard deviation
SpO_2_: Oxygen saturation
TIVA: Total intravenous anesthesia
