# Supplementary material for: Comparison of inhaled versus intravenous anesthesia for laryngoscopy and laryngeal electromyography in a rat model
Source: J Otolaryngol Head Neck Surg. 2018 Oct 20;47:64. doi: 10.1186/s40463-018-0312-9 (PMC6196000; doi:10.1186/s40463-018-0312-9)
Supplement: Supplementary file 1 — Table S1. LEMG parameters. (DOCX 61 kb) [file 40463_2018_312_MOESM1_ESM.docx]

Additional file 1: Table S1. LEMG parameters

|  | TIVA (n=9) | IA (n=19) |
| --- | --- | --- |
| Amplitude of MUP  Mean±SD, range height (Vs) | 0.004±0.005  (0.0005-0.01) | 0.005±0.006  (0.0003-0.02) |
| Amplitude of MUP  Mean±SD, range of minimal height (Vs) | 0.003±0.003  (0.0004- 0.01) | 0.004±0.004  (0.0002- 0.01) |
| Amplitude of MUP  Mean±SD, range of maximum height (Vs) | 0.01± 0.01  (0.001-0.02) | 0.01±0.01  (0.0003- 0.03) |
| Burst duration  Mean±SD, range (s) | 1.29±0.75  (0.68-3.11) | 1.03±0.24  (0.64- 1.44) |
| Burst duration  Mean±SD, range of minimal period (s) | 0.96±0.67  (0.33- 2.58) | 0.87±0.26  (0.35- 1.31) |
| Burst duration  Mean±SD, range of maximal period (s) | 1.70±1.06  (0.77- 4.33) | 1.22±0.35  (0.81- 2.25) |
| RR  Mean of ±SD, range period (s) | 1.24±0.59  (0.61- 2.61) | 1.03±0.23  (0.7061- 1.416) |
| RR  Mean±SD, range of minimal period (s) | 0.87± 0.72  (0.01-2.37) | 0.91±0.26  (0.34- 1.32) |
| RR  Mean±SD, range of maximal period(s) | 1.56± 0.67  (0.67-2.77) | 1.16±0.28  (0.74-1.60) |

MUP=motor unit potentials, Vs= volts, RR=respiratory rate, s=seconds.
